# Carbapenem alternatives for treatment of bloodstream infections due to AmpC producing enterobacterales

**DOI:** 10.1186/s12941-023-00624-9

**Published:** 2023-08-17

**Authors:** M. Ávila-Núñez, O. Lima, A. Sousa, M. Represa, P. Rubiñán, P. Celestino, M. Garrido-Ventín, L. García-Formoso, F. Vasallo-Vidal, L. Martinez-Lamas, A. Pérez-Landeiro, M Rubianes, MT. Pérez-Rodríguez

**Affiliations:** 1 Infectious Diseases Unit. Internal Medicine Department, Complexo Hospitalario Universitario de Vigo, Galicia, Spain; 2 Galicia Sur Health Research Institute (IIS Galicia Sur), SERGAS-UVIGO, Vigo, Spain; 3 Microbiology Department, Complexo Hospitalario Universitario de Vigo, Galicia, Spain; 4 Pharmacy Department, Complexo Hospitalario Universitario de Vigo, Galicia, Spain

**Keywords:** Bacteremia, Enterobacterales, AmpC-producing, Carbapenem, Cefepime, Piperacillin-tazobactam

## Abstract

**Introduction:**

Carbapenems (CR) have traditionally been the first line treatment for bacteremia caused by AmpC-producing Enterobacterales. However, CR have a high ecological impact, and carbapenem-resistant strains continue rising. Thus, other treatment alternatives like Piperacillin-Tazobactam (P-T) or Cefepime (CEF) and oral sequential therapy (OST) are being evaluated.

**Methods:**

We conducted a retrospective, single-centre observational study. All adult patients with AmpC-producing Enterobacterales bacteremia were included. The primary endpoint was clinical success defined as a composite of clinical cure, 14-day survival, and no adverse events. We evaluated the evolution of patients in whom OST was performed.

**Results:**

Seventy-seven patients were included, 22 patients in the CR group and 55 in the P-T/CEF group (37 patients received CEF and 18 P-T). The mean age of the patients was higher in the P-T/CEF group (71 years in CR group vs. 76 years in P-T/CEF group, p = 0.053). In the multivariate analysis, age ≥ 70 years (OR 0.08, 95% CI [0.007–0.966], p = 0.047) and a Charlson index ≥ 3 (OR 0.16, 95% CI [0.026–0.984], p = 0.048), were associated with a lower clinical success. Treatment with P-T/CEF was associated with higher clinical success (OR 7.75, 95% CI [1.273–47.223], p = 0.026). OST was performed in 47% of patients. This was related with a shorter in-hospital stay (OST 14 days [7–22] vs. non-OST 18 days [13–38], p = 0.005) without difference in recurrence (OST 3% vs. non-OST 5%, p = 0.999).

**Conclusions:**

Targeted treatment with P-T/CEF and OST could be safe and effective treatments for patients with AmpC-producing Enterobacterales bacteremia.

**Supplementary Information:**

The online version contains supplementary material available at 10.1186/s12941-023-00624-9.

## Introduction

Beta-lactam resistance in Enterobacterales can be produced by multiple mechanisms. The emergence of resistance is a multifactorial event due to the size of the inoculum, species involved and antibiotic concentration at the site of infection [1]. The most important is the production of beta-lactamases [2]. These include extended-spectrum beta-lactamases (ESBLs), inducible beta-lactamases or AmpC, and carbapenemases. All of them currently constitute a serious problem, due to their increasing frequency, and the important therapeutic limitations when present [3].

AmpC beta-lactamases production can be constitutive (chromosomic) or plasmid-encoded, and inducible or non-inducible. In certain bacterial species, such as *Enterobacter* spp, *Klebsiella* (formerly *Enterobacter*) *aerogenes, Serratia marcescens, Citrobacter freundii, Providencia stuartii*, and *Morganella morganii*, AmpC beta-lactamase is a constitutive and inducible gene. Therefore, all the bacteria from these species have this gene, but its expression is mediated by the exposure to certain beta-lactam antibiotics [3]. Classically, carbapenems (CR) have been the first-line treatment, especially for the treatment of severe infections caused by AmpC-producing Enterobacterales [4, 5]. However, the increasing resistance to CR, due to the spread of carbapenemase-resistant strains, and their ecological impact have resulted to looking for other alternatives.

Nowadays, there are no consensus about the best therapeutic option in AmpC-producing Enterobacterales. Though piperacillin-tazobactam (P-T) and cefepime (CEF) have been evaluated in previous studies [3, 6], last IDSA guidelines did not recommend them in patients with severe infection [7]. The aim of this study was to analyse the impact of P-T or CEF in the targeted treatment of patients with AmpC-producing Enterobacterales and safety of OST.

Another way to reduce antibiotic selection pressure is oral switching. Regarding AmpC-producing *Enterobacterales*, there are good bioavailability drugs for oral sequential therapy (OST), such as, fluoroquinolones or trimethoprim-sulfamethoxazole [8, 9]. To date, there are no published data regarding the safety of this therapeutic possibility in infections due to AmpC producers. However, there are studies with favourable results in other severe infections such as endocarditis [10], systemic infections due to *S .aureus* [11] and osteoarticular infections [12].

## Methods

We conducted a retrospective, single-centre (University Hospital Complex of Vigo) observational study. All patients above 18 years of age with bacteremia due to AmpC-producing Enterobacterales (*Enterobacter spp, Klebsiella aerogenes, Serratia marcescens, Providencia spp, Morganella morganii or Citrobacter freundii)* isolated in our hospital between March 2020 and May 2022 were included. An infectious disease specialist reviews every day the results of all blood cultures. The usual intravenous doses were: CEF 1-2 g every 8 h, P-T 4 g every 8 h, and meropenem 1 g every 8 h.

Exclusion criteria were: patients with limitation of therapeutic effort, those who died in the first 48 h after bacteremia, and the absence of information about their outcome. Those with polymicrobial bacteremia and those who received an antibiotic treatment other than CR, or P-T/CEF were also excluded.

### Variables and definitions

The primary endpoint was to evaluate the clinical success in patients with bacteremia due to AmpC-producing *Enterobacterales* treated with CR vs. P-T/CEF. Clinical success was defined as a composite outcome of clinical cure, 14-day survival, and no adverse events. Clinical cure was defined as the resolution of signs and symptoms of infection (assessed based on vital signs, SOFA score progression, and laboratory data) after 7 days of treatment. Adverse events were defined as the appearance of nephrotoxicity, encephalopathy, seizures, or hematologic toxicity, all considered secondary to antibiotic treatment by the attending physician.

Secondary outcomes were to evaluate the duration of antibiotic treatment of the different therapeutic regimens, assess the safety of OST, and to analyse the length of hospital stay in patients with OST versus those who received exclusive intravenous treatment. The safety of OST was evaluated through the recurrence rate. Recurrence rate was defined as the isolation of the same microorganism in blood cultures, within 90 days after completion of antibiotic treatment. OST was started, as targeted therapy, for those patients that had achieved clinical improvement (resolution of sepsis), that had no fever and were able to take oral medication.

We collected the patients’ clinical and epidemiological characteristics, including immunosuppression (chemotherapy, chronic steroid treatment). Being a carrier of a vascular or urinary catheter or having undergone surgery in the previous month was also recorded. Comorbidity was determined using the Charlson index [13]. Likewise, the severity of infection was classified according to the Third International Consensus Definitions for Sepsis and Septic Shock (Sepsis-3) criteria [14]. Friedman’s criteria were used to classify the origin of the infection as community-acquired, healthcare-associated or nosocomial [15]. Empirical and targeted treatment, oral switch of antimicrobials, total duration of antibiotic treatment, and length of hospital stay were also recorded.

### Microbiology

Blood cultures were processed in the Microbiology Laboratory of the University Hospital Complex of Vigo according to current regulations and procedures. Bacteremia was defined as, at least, one peripheral blood culture showed AmpC-type chromosomal beta-lactamase-producing Enterobacteriaceae (*Enterobacter* spp., *Klebsiella aerogenes*, *Citrobacter freundii*, *Morganella morganii*, *Providencia* spp., or *Serratia marcescens*). Bacterial identification at the species level was performed using the MALDI-TOF MS system (Bruker Daltonics, Bremen, Germany) and antimicrobial susceptibility tests with the VITEK2 system (bioMérieux, Marcy l’Etoile, France). The minimum inhibitory concentrations were evaluated according to the criteria of the European Committee on Antimicrobial Susceptibility Testing (EUCAST) [16]. All patients treated with CEF had strains with MIC < = 1 mg/l and those that receive P-T the MIC was < = 4 mg/l.

### Statistical analysis

The statistical package SPSS v24.0 (IBM, Chicago, IL, USA) was used for data analysis. A descriptive analysis of all the variables included in the study was performed. Quantitative variables were described as median ± interquartile range (IQR). Qualitative variables were described by their absolute and relative frequencies. Binary logistic regression analysis and inverse probability treatment of treatment weighting (IPTW) was performed to analyse the variables associated with clinical success. For the safety analysis of OST, a binary logistic regression analysis was performed.

## Results

A total of 129 patients with AmpC-producing Enterobacterales bacteremia were identified. A total of 52 patients met exclusion criteria; 18 were excluded due to polymicrobial bacteremia, 5 due to lack of clinical data, 7 due to limitation of therapeutic effort or died within the first 48 h, and 22 were excluded for receiving treatment other than CR or P-T/CEF. Finally, 77 patients were included, 22 received CR and 55 P-T/CEF (37 CEF and 18 P-T). Species included were *Serratia marcescens* (24 strains), *Enterobacter spp* (36 strains), *Citrobacter freundii* (9 strains), *Morganella morganii* (7 strains) and *Citrobacter braakii* (1 strain).

The clinical characteristics of both groups are showed in Table 1. The mean age was higher in the P-T/CEF group, (CR, 71 years [51–77] vs. P-T/CEF, 76 years [65–82], p = 0.053), with a predominance of males in both groups. Patients who received P-T/CEF had a higher rate of comorbidity (Charlson index ≥ 3, CR, 36% vs. P-T/CEF, 44%, p = 0.616). However, sepsis or septic shock (CR, 59% vs. P-T/CEF, 38%, p = 0.129) and ICU admission (CR, 32% vs. P-T/CEF, 16%, p = 0.212) were more frequent in patients treated with CR.

**Table 1:** Clinical characteristics of patients treated with carbapenem vs. cefepime/piperacillin-tazobactam

	Carbapenem (n = 22)	P-T/CEF (n = 55)	P
Age, years (IQR)	71 (51–77)	76 (65–82)	0.053
Sex, male, n (%)	16 (73)	41 (75)	0.999
Comorbidities, n (%)			
- Ischemic heart disease - Heart failure - Dementia - COPD - Diabetes mellitus - Chronic kidney failure - Solid tumour	2 (9) 2 (9) 2 (9) 2 (9) 4 (18) 3 (14) 6 (27)	9 (16) 4 (7) 6 (11) 7 (13) 8 (15) 6 (11) 17 (31)	0.497 0.999 0.999 0.712 0.734 0.709 0.999
Charlson index ≥ 3, n (%)	8 (36)	24 (44)	0.616
Acquisition type, n (%)			
- nosocomial - community - healthcare	11 (50) 10 (46) 1 (5)	30 (55) 20 (36) 5 (9)	0.803 0.606 0.668
Source of infection, n (%)			
- urinary - catheter - respiratory - abdominal - surgical wound - cutaneous - unknown	8 (36) 4 (18) 0 3 (14) 0 1 (5) 1 (5)	18 (33) 7 (13) 2 (4) 5 (9) 4 (7) 0 3(6)	0.794 0.719 0.999 0.682 0.320 0.286 0.999
Previous procedures, n (%)			
- surgery previous month - urinary catheter - vascular catheter	7 (32) 10 (46) 7 (32)	17 (31) 15 (27) 9 (16)	0.999 0.178 0.212
Sepsis, n (%)	13 (59)	21 (38)	0.129
ICU, admission, n (%)	7 (32)	9 (16)	0.212
Time until adequate treatment, mean days (IQR)	1 (1–3)	2 (1–2)	0.907
Treatment duration, mean days (IQR)			
- Intravenous days - Oral days - Total duration	10 (7–11) 0 (0) 11 (8–17)	9 (6–13) 3 (0–7) 12 (9–15)	0.383 **0.020** 0.390
OST, n (%)	4 (18)	32 (58)	**0.002**
In-hospital stay, mean days (IQR)	17 (10–41)	16 (10–22)	0.363
Adverse events, n (%)	1 (5)	1 (2)	0.492
Evolution, n (%)			
- Clinical cure - Recurrence - 14-day mortality	18 (82) 2 (9) 4 (18)	50 (91) 1 (2) 3 (6)	0.267 0.195 0.098
Clinical success	17 (77)	50 (91)	0.138

Note; CEF, cefepime; COPD, chronic obstructive pulmonary disease; ICU, intensive care unit); IQR, Interquartile Range; OST, oral sequential theraphy; P-T, piperacillin-tazobatam;

The time to adequate antibiotic therapy (CR, 1 day [1–3] vs. P-T/CEF 2 days [1, 2], p = 0.907) and the total duration of antimicrobial treatment (CR, 11 days [8–17] vs. P-T/CEF 12 days [9–15], p = 0.390) were similar in both groups.

### Clinical success

Clinical success was higher, but not significant, among patients treated with P-T/CEF (CR, 77% vs. P-T/CEF 91%, p = 0.138). Adverse events were low among both groups. Nevertheless, a higher rate of recurrence (CR, 9% vs. P-T/CEF, 2%, p = 0.195) and mortality at 14 days (CR, 18% vs. P-T/CEF, 6%, p = 0.098) was observed in the CR group.

**Fig. 1:**
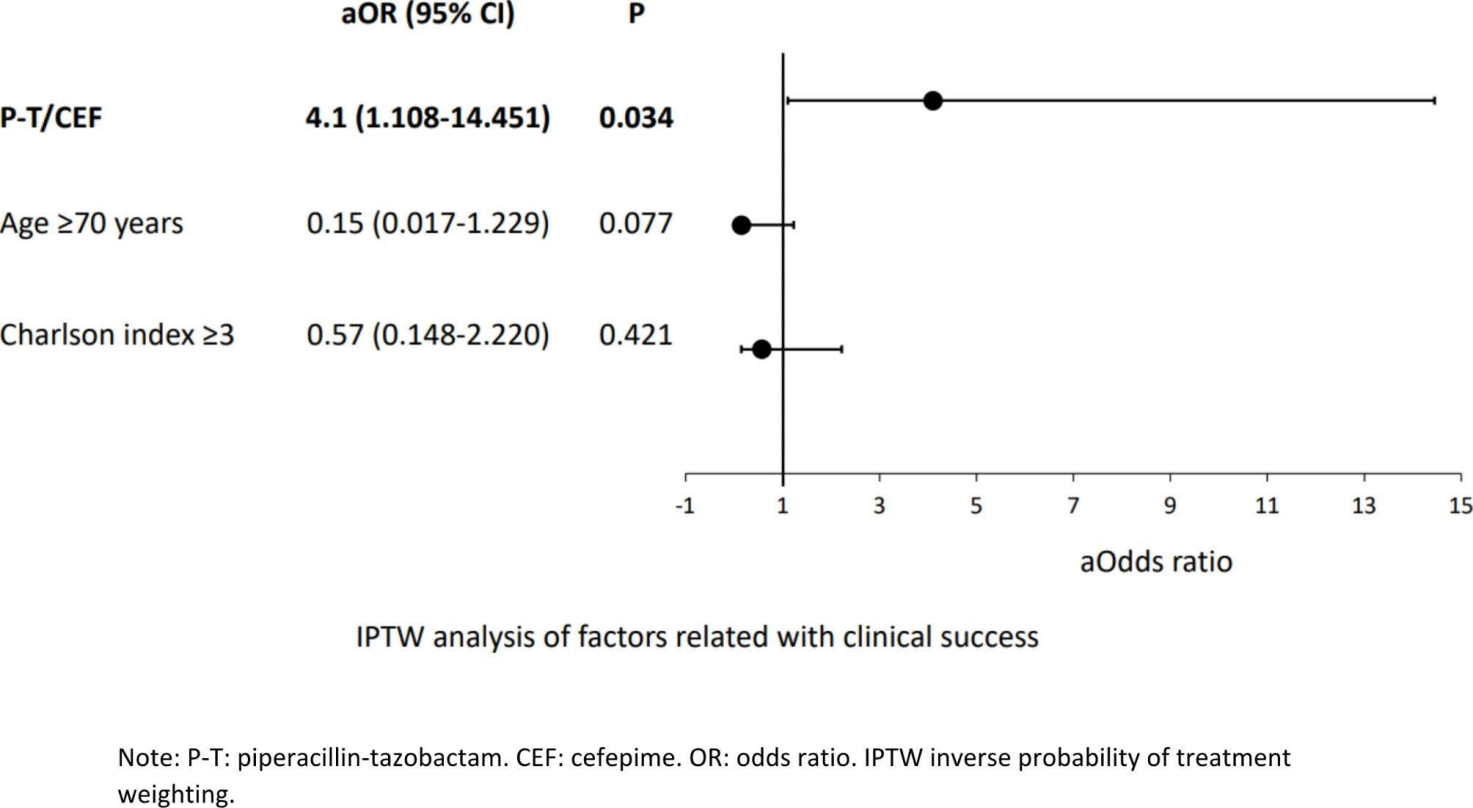
IPTW analysis of factors related with clinical success. Note: P-T: piperacillin-tazobactam. CEF: cefepime. OR: odds ratio. IPTW inverse probability of treatment weighting

In the multivariate analysis (Table 2), age ≥ 70 years (OR 0.08, 95% CI [0.007–0.966], p = 0.047), and a Charlson index ≥ 3 (OR 0.16, 95% CI [0.026–0.984], p = 0.048), were associated with a lower clinical success. On the other hand, treatment with P-T/CEF was associated with a higher clinical success (OR 7.75, 95% CI [1.273–47.223], p = 0.026). P-T/CEF, adjusted by age and comorbidities, was associated with a higher clinical success using IPTW analysis adjusted by age and comorbidities (Fig. 1). No differences were found between patients that received CEF and P-T (Supplementary material 1).

**Table 2:** Univariate and multivariate analysis of factors related to clinical success

	Clinical success (n = 67)	RR (95% CI)	p	OR (95%CI)	p
Age ≥ 70 years		0.9 (0.73–0.98)	0.083	0.08 (0.007–0.966)	0.047
- Yes - No	40 (82) 27 (96)				
Heart failure		0.8 (0.42–1.33)	0.172	0.45 (0.066–3.095)	0.419
- Yes - No	4 (67) 63 (89)				
Charlson index > 3		0.8 (0.69–1.02)	0.083	0.16 (0.026–0.984)	0.048
- Yes - No	25 (78) 42 (93)				
CEF/P-T		1.2 (0.92–1.50)	0.138	7.75 (1.273–47.223)	0.026
- Yes - No	50 (91) 17 (77)				

Note: CEF, cefepime; P-T, piperacillin-tazobatam

### Oral sequential therapy

OST was performed in 36 patients (47%). Characteristics of patients with OST and non-OST are shown in Table 3. Patients of non-OST group had a higher percentage of comorbidities (Charlson index ≥ 3, non-OST, 51% vs. OST 31%, p = 0.104) and more severe infection (sepsis, non-OST, 56% vs. OST, 31%, p = 0.038). OST was more common in patients treated with P-T/CEF (non-OST, 56% vs. OST, 89%, p = 0.002).

**Table 3:** Characteristics of patients with oral sequential treatment

	Non-OST (n = 41)	OST (n = 36)	P
Age, mean years (IQR)	71 (58–82)	75 (66–80)	0.617
Male, n (%)	24 (59)	33 (92)	**0.001**
Comorbidities, n (%)			
- ischemic heart disease - dementia - COPD - diabetes mellitus - chronic kidney failure - solid tumour	3 (7) 5 (12) 6 (15) 4 (10) 5 (12) 11 (27)	8 (22) 3 (8) 3 (8) 8 (22) 4 (11) 12 (33)	0.101 0.717 0.490 0.208 0.999 0.621
Charlson index ≥ 3, n (%)	21 (51)	11 (31)	0.104
Source of infection, n (%)			
- urinary - catheter - respiratory - abdominal - surgical wound - cutaneous - unknown	12 (29) 7 (17) 2 (5) 6 (15) 1 (2) 0 3 (7)	14 (39) 4 (11) 0 2 (6) 3 (8) 1 (3) 1 (3)	0.470 0.528 0.496 0.271 0.335 0.468 0.618
Sepsis, n (%)	23 (56)	11 (31)	**0.038**
ICU admission, n (%)	11 (27)	5 (14)	0.260
P-T/CEF, n (%)	23 (56)	32 (89)	**0.002**
Adverse events, n (%)	2 (5)	0	0.496
In-hospital stay (IQR)	18 (13–38)	14 (7–22)	**0.005**
Recurrence, n (%)	2 (5)	1 (3)	0.999

Note; CEF, cefepime; COPD, chronic obstructive pulmonary disease; ICU, intensive care unit; IQR, Interquartile Range; P-T, piperacillin-tazobatam; OST, oral sequential therapy

The duration of treatment was longer in patients with OST (non-OST, 10 days [8–14] vs. OST, 15 days [11–20], p = 0.001), but in-hospital stay was shorter (non-OST, 18 days [13–38] vs. OST 14 days [7–22], p = 0.005). The most frequently used antibiotics in OST were trimethoprim sulfamethoxazole (57%) and quinolones (35%). Finally, the recurrence of the bacteremia within 90 days after first episode was similar among the two groups of treatment (OST 3% vs. no-OST 5%, p = 0.999). None of the strains causing recurrent infection showed increase of CEF or P-T MIC.

## Discussion

The results of our work show that P-T/CEF could be a safe and effective option for the targeted treatment of AmpC-producing Enterobacterales bacteremia. Adjusted IPTW, P-T/CEF treatment showed relation with an increased clinical success.

Over the last years, some studies have shown no difference on mortality among those patients treated with P-T/CEF vs. CR [3, 17]. Even more, P-T/CEF therapy has been associated with no resistant selection [3]. Last IDSA guidelines for treatment AmpC beta-lactamase producing Enterobacterales recommend treatment with CEF, if strain MIC is < = 2 mg/l [7]. Nonetheless, P-T is only considered an appropriate treatment for patients with non-severe infection. In our study, CEF was the most common targeted treatment in patients with AmpC-producing Enterobacterales (37/55, 67%). However, we could not find any different in the efficacy among CEF and P-T treatment, as well as other authors previously [3, 17].

The main concern, about treatment of severe infection due to AmpC-producing Enterobacterales with CEF or P-T, is the selection of resistant strains. After CEF exposure, MIC can increase by up to 10 dilutions. However, appearance of resistant strains is not very common, and it is related with some species [18]. In our work, we did not detect modification in MIC in the strains of patients with recurrent infection.

Another important issue of this work is the information about safety of OST in the treatment of AmpC producing Enterobacterales bacteremia. Regarding AmpC-producing *Enterobacterales*, there are good bioavailability drugs for oral sequential therapy (OST), such as, fluoroquinolones or trimethoprim-sulfamethoxazole [8, 9]. To date, there are no published data regarding the safety of this therapeutic possibility in infections due to AmpC producers. However, there are studies with favourable results in other severe infections such as endocarditis [10], systemic infections due to *S .aureus* [11] and osteoarticular infections [12]. Some studies analyse the use of quinolones, but the route of administration is not specified [19]. In this work, ciprofloxacin or trimethoprim sulfamethoxazole were the most used drugs, principally due their high bioavailability. As in other infections or microorganisms [11, 20] patients receiving OST had lower risk of adverse events and a shorter hospital stay. Moreover, no increased recurrence rate was observed in this group of patients. Also, a systematic review, that compared quinolones or cefepime vs. carbapenems in patients with bacteremia due to AmpC-type beta-lactamase-producing Enterobacterales, did not find differences on mortality [19].

This study has several limitations. First, it is a single-centre, retrospective observational study. However, this type of design allows to have real-life information about the use of antimicrobials and their impact in the evolution of patients. Second, the number of patients included was low, and distribution among the treatment groups was not well-balanced. In order to minimise this bias, we performed IPTW. Finally, we could not determine the number of patients that received beta-lactams in extended- or continuous-infusion. This has been shown to improve clinical outcomes in some non-randomized studies [21].

Treatment with P-T/CEF could be a safe and effective treatment option in patients with bacteremia due to AmpC-producing Enterobacterales, with a similar clinical success to carbapenem. OST may be an option in patients that have an early improvement, reducing in-hospital stay and without increasing recurrence.

### Electronic supplementary material

Below is the link to the electronic supplementary material.

Supplementary Material 1

## Data Availability

Data archiving is not mandated but will be made available on reasonable request.
