# Supplementary material for: Carbapenem alternatives for treatment of bloodstream infections due to AmpC producing enterobacterales
Source: Ann Clin Microbiol Antimicrob. 2023 Aug 17;22:75. doi: 10.1186/s12941-023-00624-9 (PMC10436381; doi:10.1186/s12941-023-00624-9)
Supplement: Supplementary file 1 — Supplementary Material 1 [file 12941_2023_624_MOESM1_ESM.docx]

*Supplementary material: Clinical characteristics of patients with AmpC producing Enterobacterales treated with Piperacillin-Tazobactam and Cefepime.*

|  | P-T  (n = 18) | CEF  (n = 37) | P |
| --- | --- | --- | --- |
| Age >70 years | 13 (72) | 24 (65) | 0.761 |
| Sex, male, n (%) |  |  |  |
| Neutropenia, n (%) |  |  |  |
| Charlson index ≥3, n (%) | 6 (33) | 18 (49) | 0.387 |
| Source of infection, n (%)   - urinary - catheter - respiratory - abdominal - surgical wound - unknown | 5 (28)  1 (6)  1 (6)  7 (39)  2 (11)  2 (11) | 13 (35)  6 (16)  1 (3)  15 (41)  2 (5)  1(3) | 0.761  0.406  0.999  0.  0.590  0.247 |
| Previous procedures, n (%)   - surgery previous month - urinary catheter - vascular catheter | 7 (28)  10 (40)  8 (32) | 17 (31)  15 (27)  9 (16) | 0.999  0.302  0.143 |
| Sepsis, n (%) | 10 (56) | 11 (30) | 0.081 |
| ICU, admission, n (%) | 6 (33) | 3 (8) | **0.046** |
| Time until adequate treatment, mean days (IQR) | 1 (0-2) | 2 (1-2) | 0.127 |
| Total treatment duration, mean days (IQR) | 11 (9-15) | 12 (9-16) | **0.621** |
| In-hospital stay, mean days (IQR) | 16 (10-19) | 15 (11-25) | 0.693 |
| Clinical success | 17 (94) | 33 (89) | 0.999 |
| Evolution, n (%)   - Clinical cure - Recurrence - 14-day mortality | 17 (94)  1 (6)  0 | 33 (89)  0  3 (8) | 0.999  0.327  0.543 |
